# Volumetric Structural Magnetic Resonance Imaging Findings in Pediatric Posttraumatic Stress Disorder and Obsessive Compulsive Disorder: A Systematic Review

**DOI:** 10.3389/fpsyg.2012.00568

**Published:** 2012-12-26

**Authors:** Fatima Ahmed, Johan Ras, Soraya Seedat

**Affiliations:** ^1^Department of Psychiatry, Stellenbosch UniversityCape Town, South Africa; ^2^South African Research Chairs Initiative, Department of Psychiatry, Stellenbosch UniversityCape Town, South Africa

**Keywords:** sMRI, pediatric, anxiety, voxel-based morphometry

## Abstract

**Objectives:** Structural magnetic resonance imaging (sMRI) studies of anxiety disorders in children and adolescents are limited. Posttraumatic stress disorder (PTSD) and obsessive compulsive disorder (OCD) have been best studied in this regard. We systematically reviewed structural neuroimaging findings in pediatric PTSD and OCD. **Methods:** The literature was reviewed for all sMRI studies examining volumetric parameters using PubMed, ScienceDirect, and PsychInfo databases, with no limit on the time frame of publication. Nine studies in pediatric PTSD and six in OCD were suitable for inclusion. **Results:** Volumetric findings were inconsistent in both disorders. In PTSD, findings suggest increased as well as decreased volumes of the prefrontal cortex (PFC) and corpus callosum; whilst in OCD studies indicate volumetric increase of the putamen, with inconsistent findings for the anterior cingulate cortex (ACC) and frontal regions. **Conclusions:** Methodological differences may account for some of this inconsistency and additional volume-based studies in pediatric anxiety disorders using more uniform approaches are needed.

## Introduction

Anxiety disorders in childhood and adolescence are common and disabling (Costello et al., 2004). They often run a chronic course and are associated with the development of other disorders, such as depression, conduct disorder, or attention deficit disorder, among others (Bittner et al., 2007). Childhood is a unique period of progressive physical, behavioral, cognitive, and emotional development. The most dramatic increase in myelination, including of the corpus callosum (CC; which connects all major subdivisions of the cerebral cortex), occurs between the ages of 6 months to 3 years and continues into the third decade of life; while gray matter, and the proportion of cerebral gray matter to white matter (reflecting reductions in synaptic density and pruning), decreases progressively after age 4 (Jernigan and Sowell, 1997). Subcortical gray matter and limbic system structures (septal area, hippocampus, and amygdala) undergo an increase in volume until the third decade (Jernigan and Sowell, 1997). Imaging studies, most commonly employing functional magnetic resonance imaging (fMRI) techniques, have burgeoned in adult anxiety disorders, such as posttraumatic stress disorder (PTSD; e.g., Lanius et al., 2001; Geuze et al., 2008), generalized anxiety disorder (GAD; e.g., Hoehn-Saric et al., 2004; Whalen et al., 2008), and obsessive compulsive disorder (OCD; Adler et al., 2000). Other functional imaging techniques, such as positron emission tomography (PET; e.g., Wu et al., 1991; Rauch et al., 1996; Shin et al., 1999) and single photon emission tomography (SPECT; e.g., Lucey et al., 1997; Bremner et al., 2000; Bussato et al., 2001) have also been utilized, specifically, to probe brain metabolic abnormalities. In addition to functional imaging, structural magnetic resonance imaging (sMRI) methods have provided insights into regional brain volumetric abnormalities, particularly in adult PTSD (Stein et al., 1997; Gilbertson et al., 2002; Villarreal et al., 2002; Pederson et al., 2004) and adult OCD (Gilbert et al., 2000; Kang et al., 2004; Szeszko et al., 2004; Riffkin et al., 2005). Potential limitations of adult studies are the inclusion of patients with considerable illness duration, diagnostic comorbidity, and prior psychopharmacological treatments, variables which may all be associated with changes in gray matter volume in OCD.

Structural magnetic resonance imaging studies in youth samples provide an opportunity to recruit subjects with a relatively shorter duration of illness, fewer comorbidities, and less prior exposure to pharmacotherapeutic interventions, and an ability to track brain development and anatomy over time. While our initial intention was to review structural morphometry in all of the five major pediatric anxiety disorders (panic disorder, OCD, GAD, PTSD, and phobic disorders), preliminary screening of the literature for sMRI studies yielded relatively few pediatric studies in GAD, panic disorder, and the phobic disorders to permit a systematic review. Hence we focus here on sMRI studies in PTSD and OCD. We specifically review sMRI studies that have used volume measuring techniques in these two disorders. The use of structural MRI, one of the most commonly used techniques because of its ability to allow for the distinction of brain tissue, has not only been advantageous in helping to delineate structural differences in patients with anxiety disorders and healthy controls but also allows for the measurement of volumetric differences. Previous sMRI methods have utilized region of interest (ROI) analysis. For example, Kwon et al. (2003) used ROI analysis to measure morphological abnormalities in OCD and schizophrenia. Findings indicated significant hippocampal reductions bilaterally in patients suffering from both OCD and schizophrenia, with significant enlargement of the left amygdala in participants with OCD compared with healthy controls. There were no significant differences in thalamic volume on ROI analysis. However, some authors (Kubicki et al., 2002) believe that ROI-based MRI studies have limitations, including difficulties in demarcating structures and in evaluating certain brain regions. In addition, ROI analysis is completely manual and time consuming. In contrast to ROI approaches which reflect volumetric differences across the entire brain, Voxel-Based Morphometry (VBM) provides data on specific areas within brain structures that are maximally different between groups.

More recently, volumetric measurements using VBM or the IMAGE software package (Rasband, 1996) have replaced the primary use of ROI analysis. VBM is a modulated analysis which is used to assess between-group regional gray matter brain volume differences. VBM can identify global changes in volume and/or density of gray and white matter. Unlike ROI-based morphometric studies, VBM does not require *a priori* determination of brain areas of interest. Limitations include the risk of motion artifacts and problems with detecting differences in small regions or regions with high variance. Using an automated process, the images are segmented into gray matter, white matter, cerebrospinal fluid (CSF), and skull/scalp compartments (Ashburner and Friston, 2001). IMAGE on the other hand uses a semi-automated segmentation method to provide valid and reliable volume measurements of specific brain structures (Rasband, 1996).

The two pediatric anxiety disorders that we focus on here are PTSD and OCD. PTSD can be a highly debilitating disorder characterized by three symptom clusters, including re-experiencing aspects of the trauma, avoidance of trauma reminders, and hyperarousal symptoms. In adults, an estimated 60% of males and 50% of females will be exposed to a traumatic event in their lifetime and about 20% of these individuals will develop PTSD (Breslau et al., 1998, 1999). One sample of adolescents and young adults indicated that the overall lifetime prevalence of PTSD in the general youth population was 9.2% (Breslau et al., 1991). A national sample of adolescents (12–17 years old) indicated that 3.7% of male and 6.3% of female adolescents met full diagnostic criteria for PTSD (Kilpatrick et al., 2003). Children who experience maltreatment and develop PTSD also suffer from developmental delays that adversely affect their interpersonal and academic trajectories (Perry, 1994).

Morphometric studies in adult PTSD have demonstrated altered brain morphology compared to controls (Bremner et al., 1995; Gurvits et al., 1997; Stein et al., 1997). For example, smaller hippocampal volumes have been reported in adult combat veterans with PTSD (Bremner et al., 1995), adult PTSD secondary to child abuse (Bremner et al., 1997) and adult female survivors of childhood sexual abuse (Stein et al., 1997). This work has been extended to adolescents, with brain volumetric investigations demonstrating global gray matter volume deficits, not confined to the hippocampus, in adolescents with PTSD. Along with these global gray matter volume deficits, gray matter deficits have also been found in the anterior cingulate cortex (ACC) and frontal lobes and also in the white matter regions of the CC, (De Bellis et al., 1999, 2002a; Carrion et al., 2001). Children and adolescents with maltreatment-related PTSD show evidence of increased catecholamine and cortisol activity (De Bellis et al., 1999). In the developing brain, elevated levels of catecholamines and cortisol may lead to adverse brain development through accelerated loss of neurons (Edwards et al., 1990), delays in myelination (Dunlop et al., 1997), and/or inhibition of neurogenesis (Tanapat et al., 1998). Furthermore, stress decreases brain-derived neurotrophic factor expression (Smith et al., 1995). All of these changes can further influence brain maturation and lead to neuroanatomical changes. While studies of traumatized children have shown evidence for dysregulation of the hypothalamic-pituitary-adrenal (HPA) axis and increased cortisol secretion (De Bellis), the majority of studies in adults have shown the opposite to be true (Yehuda et al., 1995, 2000; Goenjian et al., 1996).

Obsessive compulsive disorder is a chronic and disabling disorder that is characterized by the presence of obsessions and/or compulsions (DSM-IV-TR; American Psychiatric Association, 2000). Onset of the disorder in up to 80% of the cases is before age 18. Symptoms of OCD interfere significantly with functioning and can contribute to disruption in family life and academic functioning. Previous sMRI studies of adult OCD have found brain abnormalities in several areas including the ACC, striatum, caudate nuclei, orbitofrontal cortex (OFC), amygdala, and thalamus (Valente et al., 2005; Atmaca et al., 2008; Chamberlain et al., 2008). Functional neuroimaging studies have provided evidence for involvement of the basal ganglia, thalamus, and orbitofrontal and cingular cortices (Whiteside et al., 2004). A voxel-based study by Kim et al. (2001) in adults with OCD demonstrated increased gray matter density in left anterior OFC and thalamus and decreased density in the cerebellum and left cuneus. In adolescents, Szeszko et al. (2008) found similar increases in OFC gray matter, while Gilbert et al. (2008) found decreases in ACC gray matter volume, but increases in putamen volume. The increase in putamen volume is consistent with findings by Zarei et al. (2011) in their study of adolescents.

## Methods

### Eligibility criteria

The review was restricted to studies of structural MRI in child and adolescent OCD and PTSD. In keeping with the World Health Organization’s definition of adolescence (which includes individuals in the 10–19 year age group), a study was included if all participants in that study were younger than 20 years of age, had a diagnosis of one of the above-mentioned anxiety disorders, and underwent sMRI. Both treatment-naïve subjects and subjects on treatment were included.

### Search methods

Searches were conducted using PubMed, ScienceDirect, and PsychInfo indexing services with the following key search terms: “pediatric,” “adolescent,” “child,” “sMRI,” “structural imaging,” “VBM,” and “morphometry,” crossed one by one with the terms “OCD,” and “PTSD.” Although there was no publication time limit imposed, studies matching the aforementioned search criteria were published between 1999 and September 2012. The search was confined to English language articles only.

For a more detailed look at information extracted from all relevant articles, please see Tables 1 and 2. Articles were screened by the first and second authors, Ahmed and Ras, through an inspection of the abstracts. We also checked the reference lists of the selected articles for any additional publications that may have matched our criteria.

**Table 1:** **Structural magnetic resonance imaging (sMRI) studies in PTSD**.

Name	Subjects (*n*)	M/F	Age mean/SD	Handedness right/left/mixed	Type of trauma	Mean trauma duration (Years/SD)	Comorbidity	Procedure, aim, diagnosis, treatment	Results (PTSD > controls)
De Bellis et al. (1999)	44 PTSD	25/19	12.2/2.4	41/3	PTSD secondary to sexual abuse;	2.8/2.1	MDD = 20; Dysthymia = 29; ODD = 23; ADHD = 14	MRI 1.5T	↓Cerebral and PFC, bilateral
	61 controls	36/25	12/2.3	60/1	Sexual abuse with physical abuse; Sexual abuse with witnessing domestic violence Physical abuse;	4.7/3.6 5.9/3.0		Aim: IMAGE used to show ↓ in amygdala, hippocampus, frontal and temporal lobes, and CC	amygdala, bilateral temporal lobes and CC
					Witnessing			Diagnosis: DSM-IV	
					domestic	4.5/4.0		Treatment-naïve	
					violence	5.0/2.5		
De Bellis et al. (2001)	9 PTSD 9 controls	5/4 5/4	10.6/1.6 10.5/1.6	Not stated	Not stated	3.8	MDD = 2; MDD + suicide attempt = 2; ODD = 2; ADHD = 2; SAD = 1	MRI 1.5T	Temporal, amygdala and hippocampal volumes did not differ between groups
								Aim: IMAGE to show ↓ in hippocampus in PTSD group	
								Diagnosis: DSM-IV	
								Treatment-naïve	
De Bellis et al. (2002a)	28 PTSD 66 controls	14/4 31/35	11.47/3.0 11.58/2.8	25/3 62/4	PTSD secondary to sexual abuse;	4.3/2.3	Dysthymia = 8; MDD = 1; Dysthmyia with MDD = 13; ODD = 7; ADHD = 8; SAD = 6	MRI 1.5T	↓ PFC, R temporal lobe, CC sub regions 2, 4, 5, 6, 7
				Physical abuse; Witnessing domestic violence; Sexual abuse with witnessing domestic violence			Aim: IMAGE to determine ↓ frontal, cerebral, temporal cortex, amygdala, hippocampus, and CC		
								Diagnosis: DSM-IV	
								Treatment-naïve	
De Bellis et al. (2002b)	43 PTSD 61 controls	25/18 36/25	12.2/2.4 12/2.3	40/3 60/1	Not stated	Not stated	None	MRI 1.5T	↑ STG GM volume. STG WM ↓ in PTSD > controls
								Aim: IMAGE STG volumes	
								Diagnosis: DSM-IV	
								Treatment-naïve	
De Bellis and Keshavan (2003)	61 PTSD 122 controls	31/30 62/60	11.74/2.6 11.71/2.6	57/4 117/5	Sexual abuse;	Males 3.92/2.56 Females 4.38/1.67	Dysthmia = 41; MDD = 31; ODD = 26; ADHD = 21; SAD = 6	MRI 1.5T	↑ PFC, ↓ midsaggital area of CC in boys and girls. No difference in total CC
					Domestic violence; Sexual abuse and domestic violence			Aim: IMAGE to compare sex differences in several brain regions	
								Diagnosis: DSM-IV	
								Treatment-naïve	
Thomas and De Bellis (2004)	61 PTSD 122 controls	30/31 59/62	11.74/2.6 11.74/2.5	57/4 116/5	PTSD secondary to sexual abuse;	4.2/2.2	Dysthmia = 41; MDD = 31; ODD = 26; ADHD = 21	MRI 1.5T	No significant pituitary volume differences between groups
					Physical abuse; Witnessing domestic violence; Sexual abuse with domestic violence			Aim: IMAGE to investigate pituitary volume differences.	
								Diagnosis: DSM-IV	
								Treatment-naïve	
De Bellis and Kuchibhatla (2006)	58 PTSD 98 controls 13 GAD	30/28 50/48 8/5	12/2.4 12/2.2 12.5/2.5	Not stated	PTSD secondary to sexual abuse; Witnessing domestic violence; Sexual abuse with domestic violence	Not stated	Dysthmia = 35; MDD = 30; ODD = 25; ADHD = 20 SAD = 3	MRI 1.5T	↓ Left, right and total cerebellar volumes > healthy controls
								Aim: IMAGE to compare structural changes in cerebellum and vermis between PTSD and GAD > controls	
								Diagnosis: DSM-IV	
								Treatment-naïve	
Richert et al. (2006)	23 PTSD 24 controls	13/10 Not stated	11 11	Not stated	Separation and loss; Witnessing domestic violence; Physical abuse; Sexual abuse; Physical neglect; Emotional abuse	Not stated	MDD = 3; ADHD = 3; depressive disorder = 1; social phobia = 3; SAD = 2; simple phobia = 2	MRI 1.5T	↑ GM of middle inferior and ventral regions of PFC
								Aim: BRAINIMAGE to investigate if PFC volumes differ between groups	
								Diagnosis: DSM-IV	
								Treatment-naïve	
Carrion et al. (2009)	24 PTSD 24 controls	14/10 14/10	11/2.2 11/2.7	22/2 24/0	Witnessing domestic violence; physical abuse; separation and loss; sexual abuse; physical neglect, emotional abuse	Not stated	Depression = 4; MDD = 3; social phobia = 3; ADHD = 3; SAD = 2; GAD = 2; simple phobia = 2	MRI 1.5T	↑ GM volume in superior and inferior PFC regions
								Aim: VBM to investigate aberrant PFC volumes in PTSD group	
								Diagnosis: DSM-IV	
								Treatment-naïve	

*MDD, major depressive disorder; ODD, oppositional defiant disorder; ADHD, attention deficit and hyperactivity disorder; SAD, separation anxiety disorder*.

*GAD, generalized anxiety disorder; CC, corpus callosum; PFC, prefrontal cortex; STG, superior temporal gyrus; GM, gray matter; WM, white matter*.

**Table 2:** **Structural magnetic resonance imaging studies in OCD**.

Name	Subjects (*n*)	M/F	Age (mean/SD)	Handedness right/left/Mixed	Mean OCD duration (months/SD)	Comorbidity	Procedure, aim, diagnosis, treatment	Results (OCD > controls)
Carmona et al. (2007)	18 OCD 18 controls	13/5 13/5	12.9/2.8 13.0/3.0	15/2/1 15/2/1	Not stated	None	MRI 1.5T. Aim: VBM to compare OCD brain to controls. Diagnosis: DSM-IV. Treatment: SSRIs	↓ Total GM by 5.9%, ↓ bilateral GM in frontal and cingulate regions
Gilbert et al. (2008)	10 OCD 10 siblings 10 controls	6/4 6/4 6/4	12.9/2.7 13.1/3.0 13.4/2.6	10/0 10/0 10/0	Not stated	None	MRI 1.5T. Aim: VBM to assess for ↑ GM in ACC and thalamus. ↓GM in striatum in OCD and siblings > controls. Diagnosis: DSM-IV. Treatment: Psychotropic naïve	↓ GM in left ACC ↑ GM in right putamen > siblings. No difference in thalamus or left striatum
Szeszko et al. (2008)	37 OCD 26 controls	14/23 9/17	13/2.7 13/2.6	21/1/14 17/1/8	Not stated	Comorbid anxiety = 9; Dysthymia = 3; ODD = 2; ADD = 2; ADHD = 1; Trichotillomania = 2	MRI 1.5T. Aim: VBM to assess for GM alterations in striatum, thalamus, ACC, and OFC. Diagnosis: DSM-IV. Treatment: Psychotropic naïve	↑ OFC GM. ↑ putamen volume. No increase in ACC GM
Lazaro et al. (2009)	15 OCD 15 controls	8/7 8/7	13.7/2.5 14.3/2.5	15/0 15/0	21.2/16.5	None	MRI 1.5T. Aim: VBM to assess for changes in OFC and parietal lobe	↓GM volume in parietal regions
							Diagnosis: DSM-IV. Treatment-naïve	
Lazaro et al. (2011)	27 OCD 27 controls	15/12 13/14	15.6/1.5 16.1/1.3	27/0 27/0	28.9/27.3	None	MRI 1.5T. Aim: VBM used to determine any group differences in whole brain in treated OCD patients. Diagnosis: DSM-IV. Treatment: SSRIs or Clomipramine or both	As expected, no differences in the whole brain between treated patients with OCD > controls
Zarei et al. (2011)	26 OCD 26 controls	14/12 14/12	16.6/1.5 16.5/1.4	23/3 23/3	Not stated	None	MRI 1.5T. Aim: VBM to assess if adolescents had same alterations as adults. Diagnosis: ICD-10. Treatment: SSRIs	↑ GM in bilateral caudate and right putamen

*ODD, oppositional defiant disorder; ADD, attention deficit disorder; ADHD, attention deficit and hyperactivity disorder*.

*ACC, anterior cingulate cortex; OFC, orbitofrontal cortex; GM, gray matter*.

## Results

**Figure 1:**
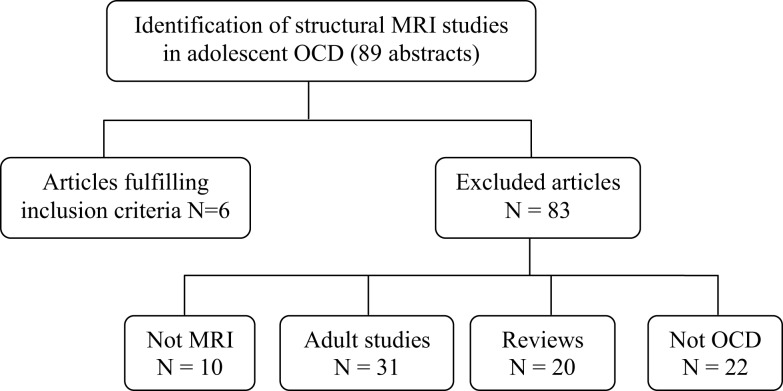
**Article selection process of structural magnetic resonance imaging studies in obsessive compulsive disorder in children and adolescents**.

**Figure 2:**
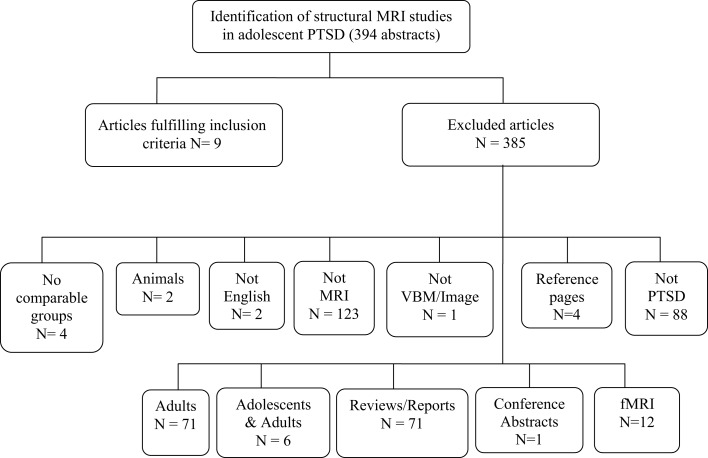
**Article selection process of structural magnetic resonance imaging studies in posttraumatic stress disorder in children and adolescents**.

We found 89 publication records that matched the search criteria for OCD. Of these 89, six studies used VBM techniques and focused on adolescents with OCD (participants 13 years and older), and were a match. We discarded the other 83 with reasons provided in Figure 1. The literature search for PTSD yielded a far greater number of publication records (*n* = 394), however, only nine of these were relevant to this review. These latter studies were all conducted in children (participants 12 years and younger); seven of these studies used the IMAGE software package for volumetric measurements, one study used the BrainImage software package (Reiss, 2002), and one used VBM methods. Figure 2 shows the reasons for exclusion of 385 articles. For a more detailed description of study and participant characteristics, please refer to Tables 1 and 2. All 15 studies included in this review used a 1.5T strength MRI scanner.

### Posttraumatic stress disorder

Table 1 summarizes the findings of PTSD studies. With the exception of one study which did not include participants with comorbid diagnoses, the other six studies included participants with various comorbidities (in particular major depressive disorder, dysthymic disorder, and attention deficit hyperactivity disorder). The aforementioned study was the only one to include mean duration of PTSD for participants. De Bellis et al. (1999) observed significantly smaller intracranial and cerebral volumes in their sample of 44 adolescents with PTSD compared with 61 controls. This was the first documented structural study of adolescent PTSD. In another study in a much smaller sample by De Bellis et al. (2001), focusing on temporal lobe, amygdala, and hippocampal volumes in nine maltreated adolescents with prepubertal PTSD and nine matched healthy, non-maltreated adolescents, no between-group volumetric differences in these structures were found at baseline, follow-up, or across time. The lack of differences might be explained by the small sample size.

Subsequent studies have highlighted both region specific and global differences in pediatric PTSD. For example, De Bellis et al. (2002b) found an increase in superior temporal gyrus (STG) volume in a sample of 43 adolescents with PTSD compared to 61 healthy matched controls. In another study, De Bellis et al. (2002a) investigated cerebral and prefrontal cortex (PFC) volumes (both gray and white matter) in 28 subjects with PTSD compared to 66 healthy, matched controls and documented a decrease in the PFC, right temporal lobe and smaller areas of the CC and its subregions (2, 4, 5, 6, and 7) in the PTSD group compared with the healthy control group. However, the same authors (2003) documented contrasting results in a study of 183 adolescents (61 with PTSD compared to 122 healthy controls). The authors found an increase in the volume of the PFC in the PTSD group, a decrease in volume in the midsagittal area of the CC but no overall differences in corpus callosal volumes in both boys and girls with PTSD. De Bellis and Kuchibhatla (2006) later went on to compare 58 participants with PTSD and 13 participants with GAD to 98 matched, healthy controls to determine any cerebellum differences between groups. They found participants with PTSD had significantly lower left, right, and total cerebellar volumes over healthy controls. Richert et al. (2006) found increased gray matter volumes of the middle inferior and ventral regions of the PFC in 23 adolescents with PTSD compared with 24 controls. Similarly, Carrion et al. (2009) found in their study of 24 adolescents with PTSD a significant increase in gray matter volume in the superior and inferior PFC compared to 24 controls. Thomas and De Bellis (2004) assessed pituitary volumes in 61 adolescents with PTSD and 121 controls and found no significant between-group differences in pituitary volumes, with pituitary volumes showing the expected increase in relation to age.

### Obsessive compulsive disorder

Table 2 summarizes findings of pediatric studies in OCD. Only one of the six studies included participants with comorbid diagnoses. The neuroanatomical regions of focus in sMRI studies of OCD have been the OFC and the striatum. The basal ganglia are also important to the underlying pathophysiology of OCD. The latter region was assessed in a study (Carmona et al., 2007) of 18 participants with OCD compared to 18 matched controls. This study showed no significant differences in gray matter volumes in the basal ganglia regions (thalamus, caudate, putamen, and globus pallidus) between the groups. However after lowering the significance threshold from *p* < 0.001 to *p* < 0.005 and re-analyzing the data to assess if age or drug treatment modulated gray matter volumes in these regions, the authors found that individuals with OCD had gray matter reductions in the frontal lobe and cingulate cortex and a total gray matter volume reduction of 5.93%. Gilbert et al. (2008) found no significant differences in the thalamus or striatum in their OCD group compared to a matched, healthy control group. However they did find significantly lower gray matter volumes in the left ACC and bilateral SFG in patients with OCD compared to healthy controls. When compared to unaffected siblings, Gilbert et al. (2008) found a significant increase in gray matter in the right putamen in patients with OCD compared to unaffected siblings. Szeszko et al. (2008) also found evidence for involvement of the putamen in adolescents with OCD (significant increase in putamen gray matter volume) and documented an increase in orbitofrontal cortical volume.

More recent work has demonstrated involvement of the parietal cortex in adolescents with OCD. Lazaro et al. (2009), using a novel longitudinal VBM approach, scanned 15 adolescents with OCD who were treatment-naïve and 15 healthy matched controls. At baseline evaluation, VBM revealed a significant decrease in gray matter volumes for the OCD group, bilaterally in the parietal cortex. However, after 6 months of treatment with selective serotonin reuptake inhibitor (SSRI) treatment and behavioral counseling for the OCD group, differences in parietal cortex volumes no longer remained significant between the groups. This concept of treatment contributing to a change in brain volume is interesting, and it is one that Lazaro et al. (2011) explored further. In a study of 27 adolescents with already-treated and stabilized OCD and 27 matched, healthy controls, the authors sought to determine if patients with OCD had structural brain abnormalities compared to the controls after stabilization on treatment. Findings supported their hypothesis that effective treatment would result in no between-group differences in whole brain. Much more recently, Zarei et al. (2011) sought to investigate whether adolescents with OCD had the same structural brain abnormalities observed in adult studies of OCD. Their study of 26 adolescents with OCD compared with 26 healthy, matched controls showed a significant increase in gray matter volumes in both the bilateral caudate and the right putamen in those with OCD.

In relation to adult studies of OCD, the studies we review here mirror some of those findings, for example the increase in OFC volumes in adolescents with OCD documented by many (Kim et al., 2001; Valente et al., 2005; Szeszko et al., 2008). However, consistent evidence for the more pronounced morphometric changes in the striatum, amygdala (Szeszko et al., 1999), and thalamus, as seen in adults with OCD have, to date, not been substantiated by adolescent studies in OCD.

## Discussion

This systematic review highlights the need for additional sMRI volumetric data in childhood anxiety disorders. Although limited, the available data on structural brain changes in children and adolescents with anxiety disorders are largely confined to PTSD and OCD. Structural neuroimaging studies in children and adolescents with OCD and PTSD have furthered our understanding of the pathophysiology of these disorders. However, high rates of comorbidity in studies of pediatric PTSD in particular confounds the interpretation of findings. In general, the studies reported here support the notion that gray matter alterations are evident in adolescents with PTSD and that these alterations include both reductions as well as increases in gray matter volume in frontal cortex.

With regards to hippocampal and amygdala gray matter volumes, findings in adolescent PTSD are not consistent with available data in adults. Studies in adolescents have not demonstrated reductions in hippocampal or amygdala volumes. A meta-analysis of hippocampal volumes in both adults with PTSD stemming from childhood maltreatment and children with maltreatment-related PTSD was conducted by Woon and Hedges (2008). They included three pediatric studies (De Bellis et al., 1999, 2001, 2002a) and four adult studies (Bremner et al., 1997, 2003; Stein et al., 1997; Pederson et al., 2004) and found no significant differences in hippocampal volumes between the PTSD group and healthy, matched controls. However, in examining the adult studies, significant hippocampal reductions were found. Medication moderated the findings in the PTSD samples and in trauma-exposed controls whereas demographic variables (e.g., age and gender) were significant moderators for the analysis of PTSD samples and non-trauma-exposed controls. The ACC was significantly smaller in adults with PTSD, and amygdala volumes bilaterally were significantly smaller in analyses of homogenous subsamples with PTSD. Although small sample sizes, sample characteristics (e.g., type of trauma, trauma severity, multiplicity of trauma, comorbidity, treatment) and methodological heterogeneity (e.g., scanning measurement differences) may partially explain this, it is also possible that structural brain changes associated with PTSD only manifest with time and may not be evident in adolescent samples where disease chronicity has not yet set in. This has potentially important implications for early intervention as well as for longer term prognosis, as effective interventions that are initiated early in trauma-exposed populations and adolescent populations may have application in preventing structural brain changes and other sequelae. On the other hand, the explanation of slowly evolving structural brain changes is inconsistent with findings that pre-trauma reduction in hippocampal volume precedes the development of PTSD (Gilbertson et al., 2002).

Findings of volumetric differences of the PFC in pediatric studies have been mixed, with some studies showing a reduction (De Bellis et al., 1999) and others an increase (Carrion et al., 2009) in gray matter volumes. Structural PFC changes are consistent with adult data demonstrating small volume changes in multiple frontal lobe and limbic system structures, including reductions in the ACC. The PFC controls many executive function tasks including planned behavior (Tanji and Hoshi, 2007), working memory (Kane and Engle, 2002), and motivation (Weinberger, 1987), and involvement of the PFC in PTSD has been well documented over the years (Shin et al., 2006). The CC is also an area that has shown significant structural changes in adolescents with PTSD when compared to healthy controls. Karl et al. (2006) conducted a meta-analysis that included three studies (one adult, two adolescent) and found that the CC was significantly smaller (medium effect size) in a primarily pediatric sample with PTSD compared to healthy controls. Jackowski et al. (2008) also showed, using DTI (Diffusion Tensor Imaging), axonal structural alterations in maltreated children with PTSD, including a reduction in myelination in the CC. This is relevant given that myelination of the CC occurs throughout childhood (Giedd et al., 1996) and early psychological trauma, with concomitant excessive release of stress hormones, may adversely affect myelination of the developing brain. De Bellis et al. (1999, 2002a) has provided further evidence of reduction in CC volume in adolescents with PTSD, specifically in mid sagittal areas of the CC. The CC normally continues to develop into adulthood, increasing in size as it develops (Keshavan et al., 2002a,b). De Bellis and Keshavan (2003), in their longitudinal study of children with PTSD (particularly male children), did not, however, find the normal age-related increase in corpus callosal size.

With regard to child and adolescent OCD, the main results of this review reveal conflicting findings, as detailed above. In comparing volumetric findings in childhood OCD with adult OCD, there are a number of similarities that deserve mention including increased basal ganglia gray matter volumes (especially in the putamen; Pujol et al., 2004; Yoo et al., 2008) and decreased gray matter volumes bilaterally in the frontal lobe and ACC (Yoo et al., 2008). These findings have not been evident across all studies. An interesting finding in both adult and pediatric studies relates to volume changes in the parietal cortex, with differences observed in the directionality of findings. For example, increased gray matter volume in the right parietal cortex has been observed in some adult studies (Radua and Mataix-Cols, 2009). In contrast, Lazaro et al. (2009) demonstrated decreased parietal gray matter volume in their pediatric study. While the parietal cortex is not a region traditionally implicated in OCD, some reports suggest that its importance may have been overlooked. The basal ganglia have long been hypothesized to play a key role in the mediation of obsessive compulsive symptoms. The conflicting findings of basal ganglia gray matter changes in pediatric OCD reviewed here are consistent with studies in adults. A recent meta-analysis (Radua and Mataix-Cols, 2009) of adult gray matter changes in OCD reported two main findings: increased bilateral regional gray matter volumes in the lenticular nucleus (mainly ventral anterior putamen) as well as decreased bilateral regional gray matter volumes in dorsal mediofrontal/anterior cingulate gyri. Findings of this study also revealed increased gray matter volumes in the right parietal cortex in individuals with OCD, further suggesting that differences may be particularly apparent in individuals with OCD without comorbid OCD. Contrary to functional neuroimaging studies of OCD implicating the OFC, the aforementioned meta-analysis failed to reveal significant differences in the OFC in adults with OCD.

The variability of findings documented may, in part, be explained by sample heterogeneity and methodological differences. OCD, of itself, is a heterogeneous disorder with complex pathophysiological underpinnings and diverse phenotypic presentations. Future studies of structural volumetric assessment in childhood OCD may benefit from more homogenous samples (e.g., based on etiological classification genetic, autoimmune, childhood adversity) and/or phenotypic classification pathological doubt, symmetry/precision, contamination, intrusive thoughts, hoarding, other [associated tics, poor insight, early onset, PANDAS (Pediatric Autoimmune Neuropsychiatric Disorders Associated with Streptococcal infections)]. Other questions that remain unanswered include whether the volumetric changes observed precede the onset of OCD symptoms (either cause or predispose) or follow the onset of OCD symptoms (consequence). Also, the specificity of the observed changes highlighted above and the relationship of OCD symptom severity [i.e., Y-BOCS (Yale-Brown Obsessive Compulsive Scale) score] to volumetric changes is, as yet, unknown and warrants investigation.

## Conclusion

Volumetric findings in pediatric OCD and PTSD are far from definitive. Methodological differences may, in part, account for this. While some progress has been made in eliciting sMRI differences in both PTSD and OCD relative to healthy controls, a number of questions remain unanswered. The influence of variables such as patient age, gender, age of trauma, type of trauma, intensity of trauma, number of trauma exposures, PTSD, and OCD duration, severity, psychotropic medication exposure, family history, and character traits (e.g., neuroticism) on structural brain changes remains unstudied. Greater sample homogeneity with regards to the above-named variables may yield larger effect sizes. In summary, additional volume-based studies in pediatric anxiety disorders using more uniform approaches are needed.

## Conflict of Interest Statement

The authors declare that the research was conducted in the absence of any commercial or financial relationships that could be construed as a potential conflict of interest.
